# Raghib Syndrome and Pulmonary Arterial Hypertension in a Pediatric Patient: Case Report and Literature Review

**DOI:** 10.3390/jcm13123623

**Published:** 2024-06-20

**Authors:** Liliana Gozar, Maria Oana Săsăran, Marius Cătălin Cosma, Daniela Toma, Andreea Georgiana Nan, Horea Gozar

**Affiliations:** 1 Department of Pediatrics 3, “George Emil Palade” University of Medicine, Pharmacy, Sciences and Technology from Târgu Mureș, Gheorghe Marinescu Street No. 38, 540136 Târgu Mureș, Romania; lili_gozar@yahoo.com (L.G.); tomadaniela94@yahoo.com (D.T.); 2 Pediatric Cardiology Clinic, Emergency Institute for Cardiovascular Diseases and Transplantation, Gheorghe Marinescu Street No. 50, 540136 Târgu Mureș, Romania; cosma.mariuscatalin@yahoo.com; 3 First Department of Psychiatry, Clinical County Hospital Mureș, Gheorghe Marinescu Street No. 38, 540136 Târgu Mureș, Romania; nandree96@yahoo.com; 4 Department of Pediatric Surgery, “George Emil Palade” University of Medicine, Pharmacy, Sciences and Technology from Târgu Mureș, Gheorghe Marinescu Street No. 38, 540136 Târgu Mureș, Romania; horea_gozar@yahoo.com

**Keywords:** Raghib syndrome, child, left superior vena cava, coronary sinus, pulmonary hypertension, atrial septal defect

## Abstract

**Background:** Raghib syndrome is a rare malformation complex consisting of the drainage of the left superior vena cava (LSVC) into the left atrium, ostial atresia of the coronary sinus and an atrial septal defect (ASD). **Case Report:** This report aims to present the case of a child newly diagnosed with Raghib syndrome, complicated by pulmonary arterial hypertension, and to review previously published cases with the same diagnosis. A six-year-old female patient presented with signs and symptoms of heart failure (Ross III), reduced exercise tolerance and severe delay in stature and ponderal development. The imagistic work-up included echocardiography, followed by computer tomography (CT) and magnetic resonance imaging (MRI), through which a diagnosis of Raghib syndrome was established, complicated by pulmonary hypertension. As in other cases presented in the literature, MRI allowed for an accurate diagnosis, detecting the absent coronary sinus. The decision regarding the surgical closure of the ASD was made, with the patient having a favorable clinical evolution but with the persistence of elevated pulmonary artery pressure, for which Sildenafil therapy was instituted. **Conclusions:** The malformation complex consisting of an atrial septal defect, ostium atresia of the coronary sinus, uncovered coronary sinus, and persistent left superior vena cava, as identified through multiple imagistic investigations, was suggestive of the rare diagnosis of Raghib syndrome in this case. Among the limited number of cases of Raghib syndrome available in the literature, the present case is distinguished by the severity of the pulmonary artery hypertension at a very young age and in the absence of other concurrent cardiac malformations.

## 1. Introduction

Raghib syndrome is an extremely rare congenital cardiac malformation, which was first described in 1965 by Raghib et al. and is characterized by a malformation complex consisting of drainage of the left superior vena cava (LSVC) into the left atrium, ostial atresia of the coronary sinus and an atrial septal defect (ASD) located in the posteroinferior angle of the atrial septum [1]. Usually, the LSVC brings deoxygenated blood into the right atrium through the coronary sinus [2]. However, the hemodynamics of Raghib syndrome are characterized by increased pulmonary flow through the ASD and a right–left shunt through the opening of the persistent left superior vena cava into an unroofed coronary sinus [3].

The clinical picture in individuals with Raghib syndrome includes fatigue, reduced exercise tolerance and syncope [4,5]. The right–left shunt constitutes a major risk factor for paradoxical embolization, whereas the persistence of the LSVC can induce and maintain atrial fibrillation [5,6]. Besides thromboembolic events, Raghib syndrome is susceptible to infectious complications, including life-threatening events such as brain abscesses and necrotizing fasciitis [2,4].

Prenatal diagnosis of persistent LSVC is crucial due to the possible association with cardiac/non-cardiac anomalies, heterotaxic syndromes and aneuploidies [7]. Perinatal management of Raghib syndrome cases can be optimized in spite of the uncertainty surrounding the adequate timing of surgical intervention [8]. The left-to-right shunt will gradually lead to right heart insufficiency and eventually to pulmonary arterial hypertension, which usually develops late during the disease’s course [9].

This report aims to present the case of a child newly diagnosed with Raghib syndrome, already complicated by pulmonary arterial hypertension. At the same time, we aim to conduct a mini-review of the literature by highlighting the particularities of currently published cases diagnosed with the same rare condition.

## 2. Case Report

We present the case of a six-year-old female patient admitted to the Pediatric Cardiology Department with signs and symptoms of heart failure (Ross III), including failure to thrive, reduced exercise tolerance and delay in neurological development. The patient’s past medical history was remarkable for repeated upper respiratory tract infections. The medical records mentioned a pediatric cardiology consultation at infant age that revealed an atrial septal defect, but the family did not present for further cardiology follow-ups. No relevant information regarding the patient’s current pathology was collected from the family medical history.

### 2.1. Clinical and EKG Findings

Clinically, the patient presented with severe weight (12.5 kg, percentile < 1) and height retardation (105 cm, percentile 2) and an increased antero-posterior chest diameter, as measured in the precordial area. Hepatomegaly was identified upon abdominal palpation (with the lower border of the liver palpable about 1.5 cm below the right costal margin), whereas a systolic murmur of grade III/VI at the pulmonary valve and a wide and fixed second heart sound with a loud P2 were audible upon auscultation. Assessment of the vital function parameters included measurement of an O2 saturation level of 96% in ambient air and an arterial blood pressure (BP) of 93/60 mm Hg. The electrocardiogram (EKG) showed a sinus rhythm, premature atrial beat (PAB) originating from the right atrium, right axial deviation, right atrial hypertrophy, right ventricular hypertrophy, and very low left ventricle voltage. The EKG Holter over 24 h recorded frequent PAB and short bursts of atrial tachycardia.

### 2.2. Diagnostic Assessment

**Figure 1:**
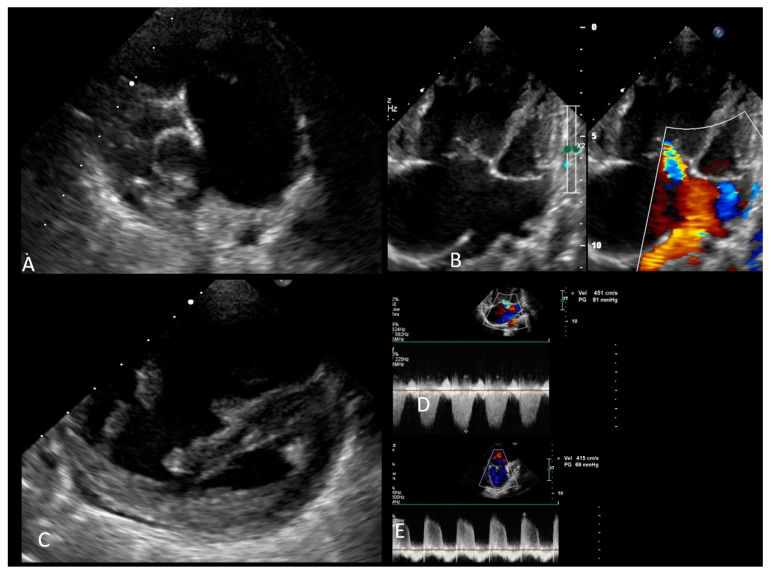
Preoperative echocardiography. (**A**) Parasternal short axis: the severely dilated PA; (**B**) 4 chamber view: large ASD, with severely dilated right atrium and right ventricle; (**C**) short axis view at the ventricles: large right ventricle and the small left ventricle; and (**D**,**E**) pulsed tricuspid and pulmonary Doppler highlights elevated pressure in the PA. Legend: ASD—atrial septal defect; PA—pulmonary artery.

Given the aforementioned clinical findings, a congenital cardiac malformation was suspected and imagistic investigations were conducted. Echocardiography (Figure 1) revealed a large ASD (ostium primum), severely dilated coronary sinus and lack of visualization of the opening of the coronary sinus in the right atrium, a bicuspid aortic valve, marked dilatation of the right heart chambers, and ultrasonographic signs of severe pulmonary hypertension. The main signs of pulmonary hypertension were the following: bulging of the interventricular septum toward the left ventricle, impressive pulmonary artery dilatation (diameter of the pulmonary artery of 3.59 cm), severe tricuspid regurgitation with a right ventricle (RV)-right atrium (RA) gradient of 80 mmHg, short pulmonary anterograde acceleration time (under 50 msec), and pulmonary valve regurgitation with a proto-diastolic gradient of 50 mmHg. The left ventricle was highly compressed (sphericity index 0.7/0.6 × 100 = 116.66) and its cavity was small in size but with a good mitral annulus size (z score mitral annulus = 1.96), whereas the apex of the heart was formed by the right ventricle.

**Figure 2:**
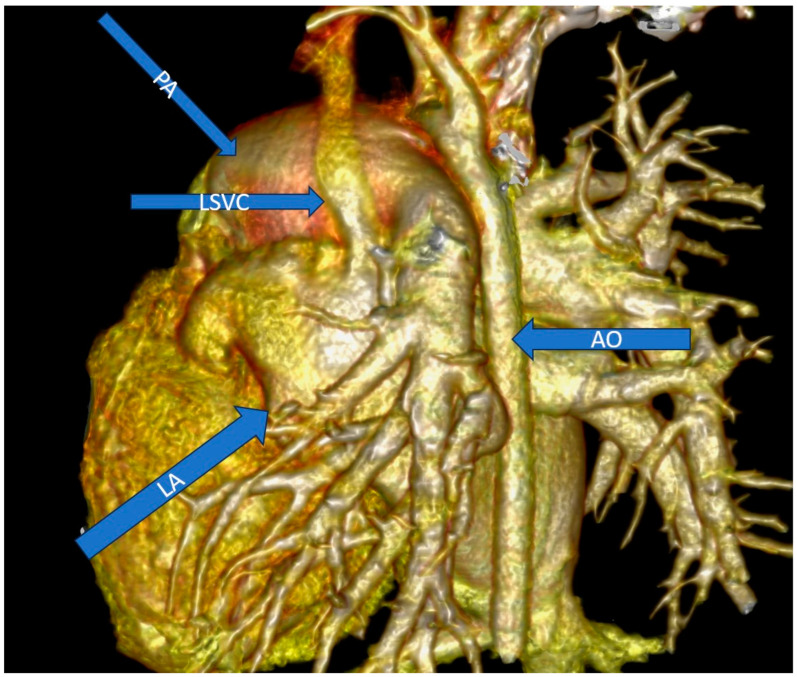
Angio CT: LSVC drains into the left atrium. Legend: AO—aorta; LA—left atrium; LSVC—left superior vena cava; PA—pulmonary artery.

We went on to perform cardiac angio-computed tomography (CT) (Figure 2), which showed an ASD of 2.6 cm, bicuspid aortic valve type I, greatly dilated right heart chambers with a right ventricle to left ventricle diameter ratio of 2.4, greatly dilated pulmonary artery with a diameter of 3.5 cm and the persistence of the left superior vena cava, opening in the left atrium.

Considering the clinical and imaging evidence that indicated the existence of severe pulmonary hypertension, we also performed exploratory cardiac catheterization in order to accurately determine the pressure in the pulmonary circulation and to establish the therapeutic indication. Cardiac catheterization revealed a mean pulmonary artery pressure of 38 mmHg and a pulmonary vascular resistance of 5 Wood units. A nitric oxide vasodilator response test was performed, and the mean pulmonary artery pressure dropped to 30 mm Hg. The exploratory cardiac catheterization revealed a wide ASD with a hemodynamically significant left–right shunt, severe pulmonary arterial hypertension with pulmonary arterial pressures, which could be reduced by nitric oxide administration, and a persistent left superior vena cava with drainage into the left atrium.

The left ventricle appeared to be smaller than normal upon echocardiographic examination. This ventricle presented a low pre-load due to the left–right atrial shunt, which also led to a decrease in the ventricular compliance. Therefore, we considered it necessary to accurately assess the left ventricular volumes and function by cardiac MRI.

**Figure 3:**
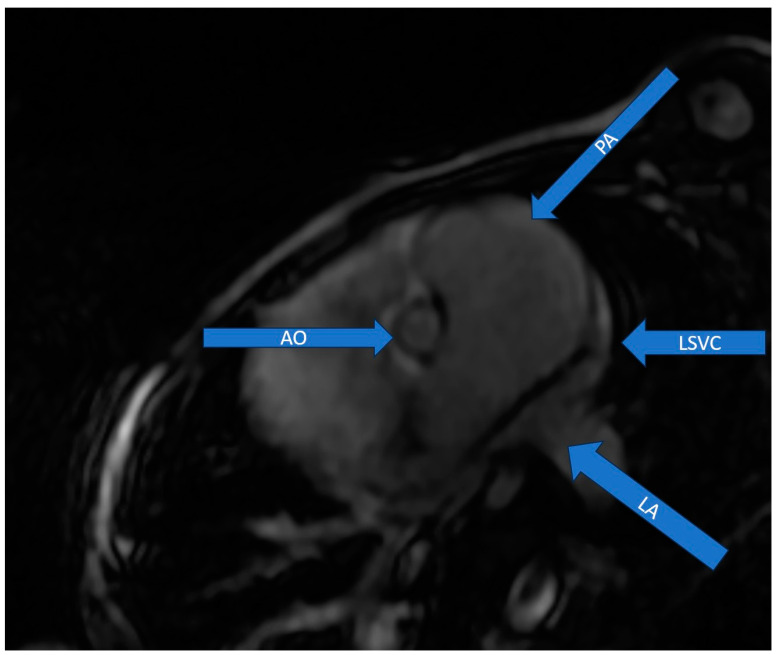
MRI: LSVC drains into the left atrium. Legend: AO—aorta; LA—left atrium; LSVC—left superior vena cava; PA—pulmonary artery.

Cardiac MRI was performed afterwards and showed an indexed left ventricular tele-diastolic volume of 62 mL/m^2^, which was interpretated as normal for the age, height and weight of the patient, together with an indexed left ventricular tele-systolic volume of 26 mL/m^2^, ejection fraction of 58% and cardiac output of 2.6 L/min. The accurate assessment of the left ventricular volume assured us that this ventricle would comply with an increase in the pre-load following surgical correction. The absence of the coronary sinus and the direct drainage of the LSVC into the left atrium were suggestive of a Raghib syndrome diagnosis (Figure 3).

### 2.3. Case Management

Considering the results of the invasive and non-invasive imaging investigations, the decision regarding the surgical closure of the ASD with a fenestrated patch was made. Median sternotomy was initially performed and visual inspection revealed a persistent left superior vena cava without an innominate vein. After right oblique atriotomy, a wide atrial septal defect of the ostium primum type was detected, whereas a common collector orifice was detected in the left atrium, draining the coronary sinus and the left superior vena cava. No abnormalities of the mitral valve were detected upon its intra-operatory inspection. Closure of the atrial septal defect with a heterologous pericardial patch was performed, leaving an 8 mm in diameter therapeutic atrial septal defect. Due to technical challenges, the left superior vena cava was not diverted toward the right atrium.

**Figure 4:**
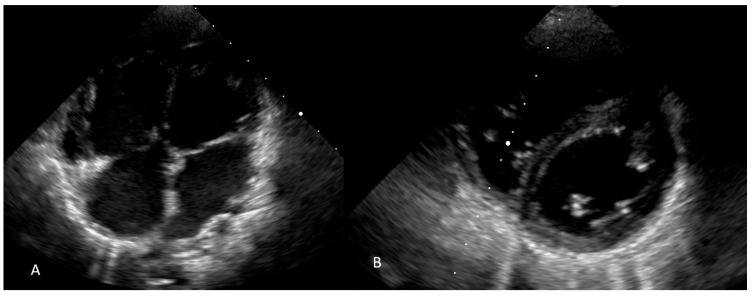
Postoperative echocardiography: (**A**) 4 chamber view; and (**B**) short axis view at the ventricles.

The postoperative evolution was slowly favorable, with the patient being extubated 2 h postoperatively, but, given the high pressures in the pulmonary circulation, the administration of a pulmonary vasodilator, namely nitric oxide, was required. The patient later had a very good clinical evolution. The postoperative echocardiography (Figure 4) revealed an increase in the LV cavity and improvement of the aortic flow, a minimal left to right shunt through the atrial septal fenestration, and reduced pulmonary pressure: the RV-RA gradient was 54 mmHg, the proto-diastolic pulmonary gradient was 22 mmHg and the end-diastolic pulmonary gradient was measured at 10 mmHg. Considering these findings, we decided to start oral therapy with Sildenafil for pulmonary hypertension, as well as with bisoprolol and spironolactone. The patient was discharged from our service on the 13th postoperative day. The patient was followed up at one, three and six months after the operation. Her clinical condition improved, as did the heart failure-associated symptoms, corresponding to the Ross II classification. The measured oxygen saturation was 93% in ambient air, but the pressure in the pulmonary artery remained above the normal values.

**Figure 5:**
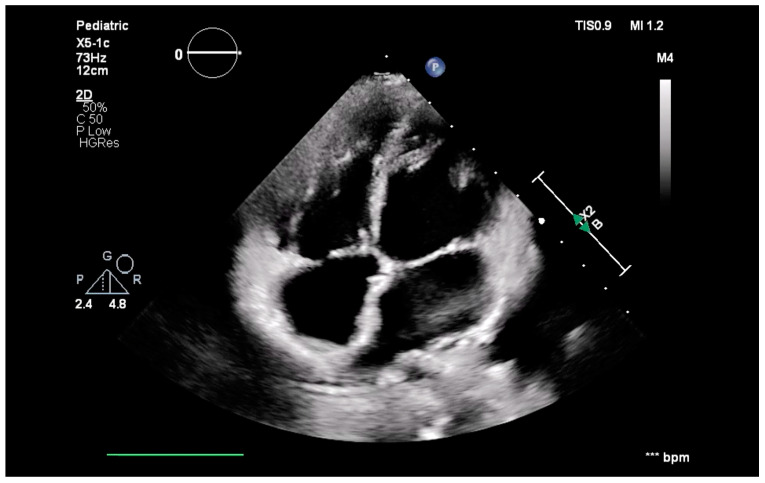
The last echocardiography, performed 1 year postoperatively; 4 chamber view.

During the most recent medical check-up of the patient, at 1 year postoperatively, the patient presented with the following: weight gain, improved tolerance to physical exertion, and O2 saturations = 97%. Echocardiographic evaluation highlighted a good biventricular contractility, normalized right heart cavities, normalized left ventricular aspect (Figure 5), rounded interventricular septum, average pressure in the pulmonary circulation of approximately 20 mmHg and an acceleration time of the anterograde flow through the pulmonary valve of 116 msec. The shunt at the atrial septum fenestration was left to right.

## 3. Literature Review

As its name states, this rare syndrome was first described by Raghib et al. in 1965, who detailed a series of eight cases in whom the left superior vena cava terminated in the left atrium [1]. In each of the five cases who were deceased, an absence of the coronary sinus was noted upon specimens obtained during autopsy. In three of these cases, an ASD was identified as well, with a particular location in the posterior-inferior angle of the atrial septum, a co-existing entity that was also described in two of the living cases. The authors suggested that a single developmental anomaly is responsible for the malformation complex (concomitant identification of a left superior vena cava terminating in the left atrium, the absence of coronary sinus and an ASD), namely, the incomplete evolution of the left atrio-venous fold [1].

Since the first description of this unique developmental complex, Lee et al. were the next ones to describe a series of three cases with coronary sinus septal defects, but a full expression of the three anomalies that the authors referred to as Raghib syndrome was only found in one of the cases, that of a 6-month-old girl with congestive heart failure [10]. An emphasis was put on the necessity of ASD closure in type II, which needs to be accompanied by a correction of the LSVC. When ligation of the LSVC cannot be accomplished, Lee et al. pointed out that the LSVC blood must be redirected toward the right atrium, either through a the creation of a baffle or through a reimplantation of LSVC into the right atrium [10]. Therefore, pre-operatory recognition of the LSVC and its aberrant drainage ensure appropriate surgical management of Raghib syndrome cases, which is mandatory for therapeutic success. Okumori et al. further described two cases of middle-aged women presenting with heart failure symptoms, in whom cardiac catheterization revealed the persistence of the LSVC, its abnormal drainage and the ASD [11]. In one of the cases, a complete surgical correction was accomplished, whereas the other patient had other associated cardiac anomalies and underwent a palliative Blalock procedure [11]. Although surgery remains the definitive treatment for Raghib syndrome, a unique case of a 58-year-old female who underwent complete correction through percutaneous repair has also been described in the literature [12].

After the case series published by Raghib et al., in which co-existing cardiac anomalies such as common atrioventricular canal and ventricular septal defect were reported, the associations between Raghib syndrome and other malformations have been subsequently described. Cor triatriatum has been by far the most frequent concurrent cardiac anomaly in patients with this malformation complex [13,14,15]. Nair et al. described a unique association between Raghib syndrome and cor triatriatum in an 18-month-old child, who had developed tachypnea early, at the age of two months, and presented pre-operatory O2 saturations of 75% in ambient air. The rapid onset of the clinical picture in this case was attributed to the restricted mitral inflow, linked to pulmonary venous hypertension and to the right ventricular dysfunction [13]. The same coexisting anomalies (cor triatriatum and Raghib syndrome) were reported in a 42-year-old female who also presented with an atrioventricular septal defect. In this case, the cor triatriatum was diagnosed intraoperatively [14]. Cor triatriatum and Raghib syndrome were also reported simultaneously with a supramitral ring (resulting in “cor tetratriatum”) and Eisenmenger syndrome in a middle-aged man [15]. Another association between Raghib syndrome and the double-orifice tricuspid valve has been reported as well, in a 42 year-old male with a history of Wolff–Parkinson–White syndrome, who was diagnosed after transthoracic and transesophageal cardiac ultrasounds [16]. The spectrum of clinical presentation and the age of diagnosis vary greatly among Raghib syndrome patients. Raghib syndrome can remain asymptomatic for years and manifest directly through a complication caused by paradoxical embolization and cryptogenic stroke, as reported in the case of 31-year-old African American female [5]. Still, very late clinical presentations have also been reported, at over 70 years of age, as exemplified in a female previously known to have permanent atrial fibrillation and pulmonary emphysema, with symptoms of heart failure [17]. In both cases, cardiac MRI played a significant role in the accurate representation of the anomaly complex [5]. Moreover, cardiac MRI also yielded an accurate diagnosis in the case of an 18-month-old boy with progressive desaturation [18].

The rarity of Raghib syndrome and its miscellaneous clinical presentations have been highlighted through the limited number of case reports/series published so far. A summary of the available literature data, previously described in further detail, has been provided in Table 1.

## 4. Discussion

The clinical picture, imaging and invasive investigations allowed us to establish the diagnosis of Raghib syndrome and pulmonary arterial hypertension in a six-year-old female patient presenting with signs and symptoms of congestive heart failure. The coronary sinus septal defect (CSSD), also known as an unroofed coronary sinus (URCS), is a very rare congenital heart defect, comprising less than 1% of ASDs and only 0.1% of all CHDs [19]. Raghib syndrome can be classified into three distinctive types depending on the persistence of the LSVC and the morphology of the coronary sinus. Type I is distinguished through a completely unroofed coronary sinus with persistent LSVC; in type II, the same unroofed coronary sinus is unaccompanied by the persistence of the LSVC; type III implies a coronary sinus with partially unroofed mid portion; whereas in type IV, the terminal portion of the coronary sinus is partially unroofed [20]. Our case is representative of Raghib syndrome type I.

Although echocardiography is widely available and accessible, and it can identify the LSVC and its drainage situs, an echocardiographic diagnosis of certainty can be challenging. A study conducted by the Tongji Medical College, Huazhong University, China, reported a 65% rate of accurate echocardiographic diagnosis through comparison between the sonographic diagnosis and post-surgical findings [19]. Unfortunately, in our case, the echocardiographic diagnosis was difficult and did not elucidate the entire malformity complex due to the particular anatomy and the difficult sonographic window caused by a thoracic asymmetry secondary to the cardiomegaly.

Cardiac computed tomography (CCT) provides much more accurate anatomical information, which, in certain circumstances, can replace echocardiography, particularly in the assessment of extracardiac vessels and coronary arteries through coronary computed tomography angiography (CCTA) [21,22]. In our case, the CT scan allowed us to make an accurate anatomical diagnosis, as well as to classify the case into its subsequent disease subtype. The study by Ai-Hua Zhi et al. demonstrated the usefulness of CT scanning for the diagnosis of unroofed coronary sinus ASD, as 100% of the patients with Raghib syndrome were diagnosed by CT scan and in only 45.6% of cases did transthoracic echocardiography help in establishing the anatomical diagnosis [23]. Moreover, Sun et al. performed a comparative study between the use of transthoracic echocardiography and computed tomography in the diagnosis of unroofed coronary sinus ASD, and they reported a diagnostic accuracy of 100% with CT and 69% with echocardiography [24]. In our case, in a similar fashion to the other literature studies cited above, computed tomography allowed an accurate description of the defect and its classification into one of the four categories.

MRI significantly aids in the localization and sizing of the CSSD and in multiplanar image acquisition, which facilitates the depiction of the LSVC and its direction flow [20]. In our case, cardiac MRI significantly helped in the measurement of the ventricular volumes and description of the coronary sinus, as happened in two previously published cases [5,18].

The spectrum of symptoms accompanying Raghib syndrome may range from asymptomatic [25] to symptoms such as shortness of breath due to the volume overload of the right ventricle, as well as symptoms suggestive of right heart failure, arrhythmias and occasionally related to pulmonary arterial hypertension [26]. Untreated unroofed coronary sinus ASD can cause pulmonary arterial hypertension secondary to intracardiac shunt, but this complication has been described in the literature more frequently in adult patients and is a rare occurrence in children [1]. Speiser et al. described the case of a 44-year-old patient presenting with signs of congestive heart failure, in whom the diagnosis of pulmonary arterial hypertension secondary to an unroofed coronary sinus ASD was established after imagistic investigations [27]. Still, the presented case is distinguished through the advanced degree of pulmonary hypertension occurring at a young age, which might not be fully explained by the intracardiac shunt at the atrial level. Severe pulmonary hypertension at a very young age has previously been reported in a 18-month-old girl with Raghib syndrome, but in this case, the underlying cause was attributed to the co-existing cor triatriatum and the restricted mitral inflow [13]. No other cases of Raghib syndrome without other associated gross cardiac malformations complicated by severe, early-onset pulmonary arterial hypertension in childhood have been reported in the literature. In the presented case, the patient had associated severe pulmonary hypertension, clinically and echocardiographically documented, and objectified by cardiac catheterization, with a mean pressure in the pulmonary circulation of 38 mmHg, reactive to the administration of nitric oxide. Considering the hemodynamics of this malformity complex, we considered on the one hand that pulmonary hypertension is secondary to the intracardiac shunt and reduced compliance of the left ventricle with a suboptimal developed systemic ventricle, and on the other hand, that the pulmonary hypertension was idiopathic. Through echocardiography, we excluded a malformation that can explain the pulmonary hypertension and an associated LSVC, such as the atrioventricular septal defect. Afterwards, we considered that a step-by-step approach is the safest for the patient.

During the postoperative evolution, the patient received pulmonary vasodilator treatment with Sildenafil and we noticed a decrease in the pressures in the pulmonary circulation, which gradually normalized. After normalization of the pulmonary pressure, treatment with Sildenafil was ceased. Considering this favorable outcome, after reducing the intracardiac shunt, we ruled out idiopathic pulmonary hypertension and concluded that the pulmonary hypertension was secondary to the intracardiac shunt.

Thus, this case is unique in terms of the secondary pulmonary hypertension in a six-year-old child diagnosed with Raghib syndrome.

Another particular aspect in our case was represented by the appearance of the left ventricle. The initial echocardiographic evaluation revealed a small left ventricle, with normal diastolic function, a non-stenotic mitral valve and a normal-sized mitral annulus. In order to accurately document the volume, function and appearance of the left ventricular myocardium, we performed a cardiac MRI with contrast material, which revealed a normal indexed end-diastolic volume (62 mL/m^2^), adequate ejection fraction of 58% and the normal structure of the ventricular myocardium. Thus, we concluded that the left ventricle was compressed by the greatly dilated right ventricle.

The premature recognition of Raghib syndrome might facilitate early therapeutic management and the prevention of complications [3,4,28]. Management of Raghib syndrome is mainly based upon surgical procedures or observation [3]. In cases with severe hypoxemia and shunt, a surgical approach is preferred [8]. Given that most cases presented in the literature improved significantly after successful surgery, even with complete cardiac rehabilitation [3], one can conclude that early intervention can positively impact the patient’s quality of life and can prevent the development of fatal complications. There are various intracardiac and extracardiac procedures that have been developed with the purpose of redirecting the LSVC flow toward the right atrium. The presence of a mitral or pulmonary inflow obstruction can negatively impact the success of intracardiac repairs [18]. Extracardiac repair methods mainly involve direct anastomosis, especially in children, because this maintains the growth potential and is associated with a good prognosis, significantly reducing the thrombotic complications [29]. Ligation of the LSVC and patch-closure of the ASD represent another common approach in the absence of other congenital heart defects [11]. Less invasive procedures, such as percutaneous closure, have also been successfully attempted [12]. In our case, the decision to perform the patch-closure of the ASD was made, with a favorable outcome. In patients with pulmonary artery hypertension, surgical repair of the ASD has rarely led to complications and has yielded a decrease in the pulmonary artery pressure, provided medical therapy was effective. Ligation of the LSVC was not possible due to absence of the innominate vein. The redirection of the LSVC toward the right atrium using a baffle would have implied an increase in the time required for surgical correction and therefore a higher risk of atrial arrythmias. Hence, the therapeutic decision at the moment of the diagnosis was to close the ASD with a fenestrated patch and to manage the pulmonary hypertension. However, in the future, a subsequent surgical intervention is planned in order to completely close the atrial septum, provided the residually elevated pulmonary pressure normalizes, and also to divert the LSVC.

## 5. Conclusions

The malformation complex consisting of an atrial septal defect, ostial atresia of the coronary sinus and a persistent left superior vena cava was suggestive of the rare diagnosis of Raghib syndrome in the case of a six-year-old female patient who presented with symptoms of congestive heart failure. Among the previous literature data, this case is distinguished by the severity of the pulmonary artery hypertension occurring at an early age and in the absence of other concurrent cardiac malformations. Surgical intervention consisting of patch-closure of the ASD was followed by a favorable postoperative evolution.

## Figures and Tables

**Table 1:** Literature review and particularities of reported Raghib syndrome cases.

	Authors	Year of Publication	No. of Cases	Clinical Picture	Particularities of the Case/Cases
1	Raghib et al. [1]	1965	Eight cases	The common clinical findings of the patients in this case series were poor weight gain or failure to thrive, cyanosis, grade I-III systolic murmur.	Case 1: Patient aged 4 years and 12 months with associated ventricular septal defect, this patient died of pulmonary infection. Case 2: A 2-year-old patient with an associated urogenital malformation, for whom atrial septal defect closure was performed, died 8 months postoperatively of a cerebral embolism. Case 3: A 5-year-old patient with atrial septal defect closure; died shortly postoperatively Case 4: An 8-year-old patient with an associated common atrioventricular canal, for whom total surgical correction was attempted but who died immediately postoperatively. Case 5: A 9-year-old patient with associated common atrioventricular canal and a double-outlet right ventricle who underwent total surgical correction but died immediately postoperatively Case 6: A 9-year-old patient diagnosed with Raghib syndrome and mitral insufficiency, in whom the atrial septal defect was successfully closed. Postoperatively, he presented with residual mitral valve insufficiency. Case 7: A patient aged 10 years with Raghib syndrome, who had an associated ventricular septal defect and pulmonary hypertension, but in whom the ventricular septal defect was successfully closed. Case 8: A patient aged 13 years and 6 months, diagnosed with Raghib syndrome, for which atrial septal defect closure was successfully performed
2	Lee et al. [10]	1979	Three cases	Case 1: Atrial fibrillation with recent onset, congestive heart failure signs and second fixed split heart sound. Case 2: Congestive heart failure signs and failure to thrive. Case 3: Signs and symptoms of congestive heart failure.	Case 1: A 48-year-old man with Raghib syndrome in whom ligation of the LSVC and baffle repair of the sinus venosus ASD were successfully conducted, with post-operatory persistence of the heart arrhythmia. Case 2: A 13-month-old girl with Raghib syndrome (ASD: 5 mm) who had an associated ventricular septal defect, right aortic arch, double-outlet right ventricle and cor triatriatum, who could not be weaned from extracorporeal circulation after cardiac surgery and subsequently deceased. Case 3: A 6-month-old girl with Raghib syndrome, accompanied by a large ventricular septal defect and patent ductus arteriosus, in whom occlusion of the LSVC and closure of the septal defects led to complete relief of symptoms.
3	Okumori et al. [11]	1982	Two cases	The two patients presented with dyspnea on exertion, pedicle edema and cyanosis, a grade 3 systolic murmur with second fixed split heart sound.	Case 1: A 42-year-old woman with no postoperative complications-no pulmonary hypertension (ASD: 3 × 4 cm) Case 2: A 41-year-old woman with associated transposition of great vessel and severe pulmonary stenosis with no pulmonary hypertension
4	Nair et al. [13]	2014	One case	The patient had minimal central cyanosis and a grade 2/6 systolic ejection murmur, whereas a second loud heart sound was audible in the left upper sternal border.	An 18-month-old female patient diagnosed with cor triatriatum and Raghib syndrome who underwent primary total correction without postoperative complications.
5	Daruwalla et al. [5]	2015	One case	Clinical onset with neurological symptoms of stroke, without cardiac findings.	A 31-year-old female-paradoxical embolization and stroke-no pulmonary hypertension
6	Egbe et al. [16]	2016	One case	The patient’s symptoms began with an episode of palpitations associated with dyspnea, and the patient was previously known to have a pre-excitation syndrome.	A 42-year-old male patient was diagnosed with Raghib syndrome, double-orifice tricuspid valve and Wolff–Parkinson–White syndrome. Ablation of the aberrant duct and primary surgical correction was performed, and the patient had no postoperative complications.
7	Perez-Caballero et al. [18]	2016	One case	The patient presented with progressive desaturations.	An 18-month-old male patient diagnosed with Raghib syndrome in whom extracardiac correction was performed, which implied anastomosis of the LSVC to the right atrial appendage
8	Baek et al. [14].	2019	One case	The patient presented with progressive dyspnea and a syncopal episode.	A 42-year-old patient diagnosed with Raghib syndrome, cor triatriatum, partial atrioventricular canal and grade III atrioventricular block, who underwent primary surgical correction and implantation of a permanent epicardial pacemaker without postoperative complications.
9	Wang et al. [12]	2020	One case	The patient presented with a pre-syncopal episode.	A 58-year-old woman with a history of recent-onset atrial fibrillation presented after a pre-syncopal episode with no pulmonary hypertension.
10	Kumar et al. [15]	2021	One case	Patient had bluish discoloration of mucous membranes, shortness of breath on exertion, clubbing in all four extremities with oxygen saturation of 82% and wheezing in the past year.	A45-year-old male-cor triatriatum, Raghib syndrome (ASD: 26.9 mm) and supramitral ring-Eisenmenger syndrome.
11	Frecentese et al. [17]	2022	One case	The patient presented in emergency conditions with signs and symptoms of heart failure NYHA class III.	A 72-year-old woman with atrial fibrillation, pulmonary emphysema and severe pulmonary hypertension.

Legend: ASD—atrial septal defect; LSVC—left superior vena cava; NYHA—New York Heart Association.

## Data Availability

The original contributions presented in this study are included in the article; further inquiries can be directed to the corresponding author.
